# Effect of PolyGlycopleX (PGX) Consumption on Blood Lipid Profiles in Healthy, Low CVD Risk Overweight Adults

**DOI:** 10.3390/nu11040717

**Published:** 2019-03-27

**Authors:** Vicky A. Solah, Deborah A. Kerr, Deasy Irawati, Wendy Hunt, Xingqiong Meng, Roland J. Gahler, Haelee K. Fenton, Stuart K. Johnson, Anthony P. James, Simon Wood

**Affiliations:** 1 School of Molecular and Life Sciences, Faculty of Science and Engineering, Curtin University, Perth, WA 6845, Australia; h.fenton@curtin.edu.au (H.K.F.); s.johnson@curtin.edu.au (S.K.J.); 2 School of Public Health, Faculty of Health Sciences, Curtin University, Perth, WA 6845, Australia; d.kerr@curtin.edu.au (D.A.K.); deasy15ira@yahoo.com (D.I.); Wendy.Hunt@aegic.org.au (W.H.); T.P.James@curtin.edu.au (A.P.J.); simonwood@shaw.ca (S.W.); 3 Faculty of Medicine, Mataram University, West Nusa Tenggara 83125, Indonesia; 4 Australian Export Grains Innovation Centre, South Perth, WA 6151, Australia; 5 Flinders Centre for Epidemiology and Biostatistics, College of Medicine and Public Health, Flinders University, Adelaide, SA 5001, Australia; Rosie.Meng@sa.gov.au; 6 Factors Group R & D, Burnaby, BC V3N4S9, Canada; rgahler@naturalfactors.com; 7 Curtin Health Innovation Research Institute, Faculty of Health Sciences, Curtin University, Perth, WA 6845, Australia; 8 InovoBiologic Inc., Calgary, AB Y2N4Y7, Canada; 9 Food, Nutrition and Health Program, University of British Columbia, Vancouver, BC V6T1Z4, Canada

**Keywords:** blood lipids, cardiovascular, disease risk, cholesterol

## Abstract

Raised blood lipid levels are associated with a risk of a cardiovascular disease (CVD). Moderate reductions in several CVD factors such as total, low-density lipoprotein (LDL) cholesterol and non-high-density lipoprotein (non-HDL) cholesterol concentrations may be more effective in reducing overall risk than a major reduction in just one. A blind, randomised controlled trial was conducted with 120 healthy overweight (BMI 25–30) adults aged 25–70 years who were non-smokers, not diabetic and of low risk of cardiovascular disease, as assessed by the Framingham risk equation. Participants consumed 4.5 g PolyGlycopleX (PGX) as softgel capsules (PGXS) or 5 g PGX granules (PGXG) or 5 g rice flour (RF) with meals three times a day for 12 weeks. Total, LDL and non-HDL cholesterol were all significantly reduced (−6%, −5% and −3.5%, respectively) post the PGX granule treatment; however, PGX in softgel capsule form did not affect blood lipid profiles. Daily consumption of PGX granules in overweight low CVD risk adults produced lipid changes indicating a CVD preventative benefit.

## 1. Introduction

Cardiovascular disease (CVD) is the leading cause of death globally and accounted for 17.9 million deaths worldwide in 2016 [1]. The CVD risk factors that are included in the Framingham risk equation are age, gender, smoking status, diabetes, systolic blood pressure (SBP) and the ratio of total cholesterol to high-density lipoprotein cholesterol (TC:HDL). The National Vascular Disease Prevention Alliance [2] states that raised blood lipid levels have an association with risk of CVD events, and that moderate reductions in several risk factors may be more effective in reducing overall CVD risk than a major reduction in a single factor [3]. For example, significant improvements in TC, low-density lipoprotein (LDL) cholesterol concentrations and waist circumference are critical components to reducing CVD burden [4]. Lowering LDL cholesterol concentration is a primary target for reducing CVD risk, but concentrations of non-high-density lipoprotein (non-HDL) cholesterol and apolipoprotein B are now also considered important targets [5]. 

Dietary fibre (DF) intake is protective against obesity and a range of chronic disorders, including CVD, type 2 diabetes mellitus (T2D) and colon cancer [6,7,8]. DF primarily consists of non-starch polysaccharides, many of which are characterised by their ability to form viscous dispersions and/or gels in water [9]. For example, the consumption of β-glucan, a viscous soluble fibre from barley, can lead to a significant reduction in total cholesterol as well as LDL cholesterol levels [10,11]). The viscous soluble fibre in psyllium also reduces total and LDL cholesterol concentrations [12,13]. There are few reports on the effect of consumption of viscous fibres on non-HDL cholesterol, with recent reports on the intake of barley and oat β-glucan and konjac glucomannan significantly reducing LDL cholesterol and non-HDL cholesterol levels [14,15,16]. Cantero et al. [17] found insoluble fibre consumption (7.5 g/day) improved the fatty liver index (FLI) in a study of 70 subjects with metabolic syndrome. In a randomised, double-blind placebo-controlled study of 88 subjects consuming PolyGlycopleX (PGX) granules over three weeks, Carabin et al. [18] found no change from the baseline, and no significant differences between PGX and control groups for aspartate aminotransferase (AST) and alanine aminotransferase (ALT). In contrast, Turck et al. [19] reviewed a possible toxic effect based on the findings of Matulka et al. [20], who found a high level of AST and ALT in only the female Sprague Dawley rats who consumed PGX at 5% of their diet. However, Matulka et al. [20] concluded that the results were within historical control values, did not correlate with any histopathological changes and were not considered adverse. 

Several mechanisms have been proposed to explain the beneficial effects of DF on blood lipids, including short chain fatty acid (SCFA) production in the colon, reduced fatty acid synthesis, lowered body weight with fat loss, improved glycaemic control, microbial modulation, reduced inflammation and formation of non-covalent bonds with other dietary components to adsorb or entrap them [21,22]. Gaining a better understanding of the beneficial influence of increased DF intake on the multiple markers for CVD risk is an important area of preventative health research.

PolyGlycopleX (PGX) is a high-viscosity non-starch polysaccharide complex that can be consumed as a granular powder or in soft gel capsules. Consumption of PGX (a listed medicine in Australia as Alginate-Konjac-Xanthan Polysaccharide Complex, InovoBiologic, Calgary, Canada) as granules has been found to have a beneficial effect on blood lipid profiles in otherwise healthy but overweight and obese subjects [13,18,23]. Specifically, individuals consuming PGX for 21 days significantly decreased their fasting TC levels (from 4.88 to 4.20 mmol/L) and LDL cholesterol levels (from 2.89 to support the potential of PGX to contribute to the reduction of CVD risk in subjects with elevated blood lipid levels. 2.41 mmol/L) compared to the control group [18]. In a study of obese adults (mean BMI 33.0 kg/m^2^) who consumed PGX over 14 weeks and received dietary counselling, fasting TC significantly dropped from 5.69 to 4.60 mmol/L, and insulin levels decreased from 12.87 to 9.37 μU/mL [23]. Similarly, after a three-month study of obese participants, Pal et al. [13] found PGX granules significantly reduced TC concentration by 8% (~0.4 mmol/L) and decreased LDL cholesterol concentration by 13.9% (~0.17 mmol/L) in the PGX granules group compared to the control group. Pal et al. [13] did not assess PGX in softgel capsules. In addition, Lyon et al. [24] found a decrease in TC concentration in women who consumed PGX with significant reductions in both HDL and LDL cholesterol. Furthermore, a study of overweight participants (mean BMI 28.4 kg/m^2^) in which PGX was consumed in a granular form at meal time over 12 weeks, reported a significant reduction in waist circumference (2.5 cm; *p* = 0.003) [25] compared to the baseline. Collectively, these findings

Since reductions in a range of blood lipid measures may be more effective in reducing overall CVD risk than a large reduction in a single measure (such as total cholesterol), this research aimed to investigate whether PGX consumption induced changes in fasting blood lipid profiles, especially non-HDL cholesterol and apolipoprotein B48, which could be important new targets set for reduction of CVD risk. A secondary aim of this study was to determine if AST and ALT levels were in the normal range for human adults after consumption of PGX over 12 weeks. The current intervention was needed to compare for the first time the consumption of PGX granules and softgel capsules in healthy adults at low risk of CVD. 

## 2. Materials and Methods

### 2.1. Study Participants

The study recruited 120 healthy overweight (BMI 25–30 kg/m^2^) adults aged 25–70 years who were non-smokers, not diabetic and not on medication for cholesterol or blood pressure. Of the study participants, 28 were male and 92 were female. All subjects had a TC:HDL ratio less than 6. According to the Framingham equation, risk of CVD was determined as low for 98% of participants in the study. Only one participant had a moderate risk of CVD, and the elevated risk was associated with being male and over 65, not their smoking status, TC:HDL ratio or blood pressure [2]. Risk was calculated from the Absolute Cardiovascular Disease Risk Management Quick Reference Guide for Health Professionals by the National Stroke Foundation [2].

Prior to the commencement of supplementation and during the 12th week of the study, participants took before and after images of all foods and drinks (excluding water) consumed over four consecutive days using a mobile food record app (mFR) [25]. Food consumption during the entire study was ad libitum.

### 2.2. Trial Design

A blinded, randomised controlled trial was approved by the Human Research Ethics Committee, Curtin University (reference HR170/2014) and conducted at Curtin University between August 2014 and April 2016. All participants were informed on the nature of the participation, and signed informed consent was obtained before starting the study. Participants (*n* = 120) were randomly assigned into one of three parallel study groups: (1) consuming six softgel capsules containing 4.5 g of PGX (PGXS), (2) consuming 5 g PGX granules (PGXG) and (3) consuming 5 g of the rice flour (RF) control; all were consumed three times per day for 12 weeks. Participants were provided with a carry bag labelled with a three-digit code containing a 12-week supply of PGXG or RF as 5 g individual doses in identical foil sachets or three plain white jars labelled with a three-digit code, each containing one month’s supply of PGXS. Research staff were blinded to the treatment allocation until all analyses were completed. Participants reported their daily intake of the product and returned unused products so compliance could be checked. Fasting venous blood samples were collected at the baseline and after 12 weeks of supplementation. The trial was registered with the Australian New Zealand Clinical Trials Registry (reference ACTRN12614000701628).

### 2.3. Sample Collection and Analysis 

Following an overnight fast for at least 10 hours, participants were admitted to the research unit. Fasting venous blood samples were collected into BD Vacutainer EDTA tubes (Becton Dickinson, Franklin Lakes, NJ, USA) for plasma samples, and BD Vacutainer serum tubes (Becton Dickinson, Franklin Lakes, NJ, USA) for serum samples. Fasting serum samples were isolated by low-speed centrifugation after being left to clot for 30 minutes. Plasma samples were isolated following low-speed centrifugation immediately after collection. All samples were then stored in aliquots at −80 °C prior to analysis.

The fasting blood parameters of triglycerides (TG) (mmol/L), total cholesterol (TC) (mmol/L), high-density lipoproteins (HDL) (mmol/L), apolipoprotein (apo) B48 (µg/mL), aspartate aminotransferase (AST) (U/L) and alanine aminotransferase (ALT) (U/L) were measured at the baseline and week 12. LDL cholesterol and non-HDL cholesterol concentrations were subsequently calculated [26,27]. 

Chylomicron concentration was measured by determining apoB48 concentration using a commercial sandwich ELISA method with a monoclonal antibody raised against the C-terminal region of apoB48 (Shibayagi Human apoB48 ELISA Kit, Ishihara, Shibukawa, Japan; CV < 3%). This method has been validated by Kinoshita et al. [28].

### 2.4. Statistical Analysis

Per-protocol analyses with a mixed model effect were performed and reported. The outcome variables were TG, TC, HDL cholesterol, LDL cholesterol, non-HDL-cholesterol, apoB48, AST and ALT. 

A mixed effect model with clustering of participants was used to assess outcome variables for (1) within-group difference over time, and (2) between group differences in change over time. Where group differences in change over time were found, regressions using transformed values (normally distributed) were further investigated for differences across the three study groups with the Bonferroni adjustment for multiple comparisons. 

For per-protocol analysis, only those who completed the study at week 12 were included. All tests were two-tailed and a *p* value < 0.05 was regarded as statistically significant. All analyses were performed using Stata MP 14.1 (Stata Corp., College Station, TX, USA). 

## 3. Results

The subject characteristics of the participants in each study group are shown in Table 1. On average they were overweight (mean BMI 29.1 kg/m^2^). There were no significant differences between the three study groups for baseline height, weight, waist circumference or BMI. Of the 120 participants recruited and included in the initial randomised block study design (Table 1), 74 completed the 12-week duration of the study (PGXS, PGXG and RF in Table 2). The main reasons for withdrawal were due to stomach upsets and diarrhoea after PGXG consumption (attrition = 6), due to diarrhoea, headaches and difficulty swallowing softgels after PGXS consumption (attrition 6) and due to constipation and feeling ill after RF consumption (attrition = 15). 

### 3.1. Blood Profiles

Participants in the study presented as healthy, normoglycaemic, normolipidemic individuals. The fasting blood parameters glucose (mmol/L), insulin (µU/mL), triglycerides (TG) mmol/L, TC (mmol/L), HDL cholesterol (mmol/L), LDL cholesterol (mmol/L), non-LDL cholesterol (mmol/L), apoB48 (µg/mL), AST (U/L) and ALT (U/L) at the baseline and week 12 are presented in Table 2.

Within-group differences over time for each outcome variable are presented above. However, there were no significant between-group differences in change over time between the PGXS, PGXG and RF treatments.

### 3.2. Total, LDL, HDL and Non-HDL Cholesterol and TG

The mean baseline total cholesterol (TC) of the three intervention groups classified them as normal (5–5.4 mmol/L) [2,29]. Individuals with TC greater than 7.5 mmol/L are said to be at an increased risk of CVD [2]. There was a significant reduction of 6% (*p* = 0.01) in TC in the PGXG treatment group from the baseline to 12 weeks. No significant change was observed in TC level in the PGXS or RF groups. 

Mean LDL cholesterol concentration was in the healthy range of 3–3.5 mmol/L in each treatment group at the baseline [5]. There was a significant 5% decrease in LDL cholesterol concentration of 0.16 mmol/L (*p* = 0.037) in the PGXG treatment group from the baseline to 12 weeks. No significant change in LDL cholesterol was observed in the PGXS and RF groups. There is a therapeutic benefit to lowering LDL cholesterol to levels substantially below 2.5 mmol/L in high-risk individuals with existing coronary heart disease (CHD) [3]; therefore, the consumption of PGX granules has benefits for high-risk individuals as well as the low-risk individuals in this study.

At the baseline the non-HDL cholesterol mean values for each treatment group were in the normal range of 3.5–4.0 mmol/L [5]. In the PGXG treatment group, non-HDL cholesterol decreased significantly 3.5% (*p* = 0.035). No significant change was noted in non-HDL levels in the PGXS nor the RF group.

Mean TG concentrations at the baseline were in the healthy range (1.0–1.2 mmol/L) for each treatment group [30]. There were no significant reductions in TG in any group however in the PGXS group TG increased significantly (*p* = 0.03) although the final TG was still in the healthy range.

### 3.3. Apolipoprotein B48 (apoB48)

ApoB48 concentrations did not significantly change from the baseline (*p* < 0.05) after 12 weeks consumption of PGXS, PGXG or RF. A non-significant reduction was found in apoB48 in the PGX group (full data not shown) of −1.6 µg/mL (95% confidence interval (CI) −4.094 to 0.997 µg/mL) compared to RF group, and in the PGXS group of −2.0 µg/mL (95% CI −4.634 to 0.606 µg/mL) compared to RF group (Bonferroni’s multiple comparison).There were no significant within-group changes in apoB48 between 0 and 12 weeks for any of the treatments.

### 3.4. Aspartate Aminotransferase (AST) and Alanine Aminotransferase (ALT)

A significant decrease (*p* = 0.014) in AST was seen in the PGXG group, but no significant change was found in the PGXS or RF groups. ALT activity was found to decrease in the PGXG group (*p* = 0.006) as well as in the RF group (*p* = 0.03). However, it significantly increased in the PGXS group (*p* = 0.05), while remaining in the normal range of <30 U/L at 12 weeks of intervention [31].

## 4. Discussion

### 4.1. Total, LDL and Non-HDL Cholesterol 

The target for “healthy” total cholesterol concentration has traditionally been <5 mmol/L, but more recently this value has been revised to <4 mmol/L and this reduced level is recommended by the National Vascular Disease Prevention Alliance [2,32]. Therefore, in this cohort of overweight participants, treatment to induce a clinically significant reduction in TC is warranted. The 6% reduction in TC (minus 0.31 mmol/L) in the PGXG group is of a magnitude that is considered clinically important for CVD prevention [2]. Carabin et al. [18] and Pal et al. [13] also found a significant lowering of TC (0.4 mmol/L) in their studies on PGX consumption. Lyon and Reichert [23], however, reported a greater decrease of 1.0 mmol/L when the PGXG treatment was combined with a weight-loss program. The mechanism for TC reduction by PGXG may be the binding between it and bile acids in the gastrointestinal tract, which may facilitate the elimination of bile salts in the faeces [33]. This would result in the liver using more endogenous cholesterol to replenish the bile acids pool, thus lowering the concentration of circulating cholesterol [33].

Systematic review and meta-analysis of relevant research on the effects of oat, barley β-glucan and konjac glucomannan consumption [14,15,16] showed a significant reduction in LDL and non-HDL cholesterol concentration in mostly hypercholesterolemic adults. The European Food Safety Authority (EFSA) panel considers lowering blood LDL cholesterol concentrations to be physiologically beneficial in reducing CHD risk [8], and therefore the 5% LDL reduction (−0.16 mmol/L) as a result of consuming PGXG over 12 weeks found in this study may contribute to reduced CHD risk. This reduction of 0.03 mmol/L per g of PGX is similar to the reported decrease following consumption of oat and barley β-glucan by 0.04 mmol/L per g [14,15]. The reduction in average non-HDL concentration of 0.13 mmol/L found after PGX consumption is similar to the reported decrease following consumption of oat and konjac glucomannan by 0.2 and 0.1 mmol/L, respectively [14,16].

The consumption of PGX in softgel form appears not to have had a beneficial effect on CVD risk factors in the present study. Previous research on PGX in this dosage form has not investigated blood lipids, but Brand Miller et al. [34] found PGX in capsule form did not reduce acute glycaemia, and Jenkins et al. [35] found viscous fibre in softgel capsule form did not improve glucose tolerance. In a study on the effects of granules on satiety by Solah et al. [36], the importance of hydration to induce viscosity and gel formation in soluble dietary fibre formulations such as PGX was highlighted. Therefore the potential and the delay in hydration of PGX in the gastrointestinal tract due to the protective soft gel capsule may have led to the PGXS not being as effective on blood lipids as the PGXG.

The significant improvement in total, LDL and non-HDL cholesterol over 12-weeks found as a result of PGXG consumption in this study indicates its potential to reduce CVD risk. In addition, the significant reduction in waist circumference (−2.5 cm; *p* = 0.003) previously reported [25] for subjects who consumed PGXG further supports the positive health effect of its consumption. These findings highlight the beneficial effect of PGX consumption on multiple CVD risk factors in the group of lower CVD risk adult participants.

### 4.2. Apolipoprotein B48 (apoB48)

The individual concentration of apoB48 was highly variable at the baseline, ranging from 2.4 to 26.9 µg/mL. There were six participants with results >20 µg/mL apoB48. In comparison, individual concentration of apoB48 reported in Japanese subjects by Sakai et al. [37] ranged from 3.7 to 5.4 µg/mL, but there could be ethnicity factors contributing to this narrow range in the Japanese study compared to the wider range observed in our study. 

Elevated concentrations of chylomicron particles have been observed in subjects with visceral obesity [38] and metabolic syndrome [39], however few studies have examined the effect of diet and lifestyle interventions on fasting chylomicron concentrations. In the present study we did not observe any significant changes in apoB48 concentrations either within each treatment group or between groups, however a trend towards a significant reduction (−23%) was observed in the PGXG group compared to the RF group.

### 4.3. Aspartate Aminotransferase and Alanine Amino Transferase

AST and ALT enzyme activities are commonly used to assess liver function, and values in serum less than 30 U/L are considered healthy [31] (although levels less than 50 U/L have also been quoted as normal [40]). The mean values of AST and ALT activity found in all groups in the present study were in the healthy ranges at both at the baseline and after 12 weeks, despite the increase in ALT levels between week 0 and week 12 for the PGXS group. As in the present study, previous research investigating the influence of PGXG [18] reported no statistically significant change to AST and ALT serum levels after 21 days consumption (values were not reported or discussed) [18]. Although PGXS consumption increased ALT to 28.4 U/L on average, this value is considered low and in the healthy range. AST after PGXS consumption did not change. In the present study there was no adverse effect of PGXG consumption on AST and ALT, with both the baseline (24 and 24 U/L, respectively) and 12-week consumption (20 and 18.8 U/L, respectively) values being in the normal range [31].

## 5. Limitations

Recruitment required a continual sustained effort, and while we aimed to recruit for 12 months, recruitment continued for 21 months to enlist 120 eligible participants. Despite continual effort, fewer men than women volunteered for the study. Consumption of test products were self-administered and misreporting of intake may have occurred. While the absence of differences between the treatment groups (PGXS, PGXG and RF) is a limitation of the study, we feel the within-group change over time is important. Longer-term supplementation would have benefited the study.

## 6. Conclusions

Lowering total cholesterol and LDL cholesterol is a well-known primary target for reducing CVD risk; however, lowering non-HDL cholesterol and apolipoprotein B are more recent guideline inclusions. This research adds to current knowledge on the effects of PGX consumption on total, LDL and HDL cholesterol and TG and provides new knowledge on non-HDL levels. Even with the limited number of participants in this study we were able to report several important findings, specifically that daily PGXG (granules) intake resulted in reduced total, LDL and non-HDL cholesterol concentrations in overweight low risk adults. These observed moderate reductions in several risk factors may be effective in reducing overall CVD risk, indicating a possible preventative health benefit of PGX granule consumption. PGXS (soft gel capsules) was not as effective in reducing total cholesterol, LDL and non-HDL cholesterol concentrations. PGXG and PGXS consumption resulted in AST and ALT levels remaining in the normal range after 12 weeks of consumption. Further research in a larger group of normoglycaemic and normolipidaemic overweight individuals is needed.

## Figures and Tables

**Table 1:** Characteristics of all recruited study participants randomised at the baseline (*n* = 120) comparing groups.

All Participants	PGXS (*n* = 40)	PGXG (*n* = 40)	RF (*n* = 40)
Men	9	10	9
Women	31	30	31
Mean ± SD			
Age (years)	42.2 ± 16.0	46.5 ± 14.0	43.3 ± 16.8
Height (cm)	167.4 ± 9.1	167.3 ± 9.0	166.4 ± 7.9
Weight (kg)	82.7 ± 16.8	80.9 ± 16.6	81.3 ± 17.7
Waist (cm)	89.8 ± 12.8	90.7 ± 12.1	88.4 ± 14.3
Body mass index (BMI, kg/m^2^)	29.4 ± 4.8	28.7 ± 4.4	29.2 ± 4.8

**Table 2:** Blood profiles for study participants at week 0 and week 12.

Fasting Concentrations	PGXS (*n* = 27)	PGXG (*n* = 32)	RF (*n* = 15)
	Mean (SD)	Mean (SD)	Mean (SD)
Glucose mmol/L			
Week 0	5.0 (0.37)	5.2 (0.40)	5.4 (0.70)
Week 12	5.1 (0.50)	5.3 (0.30)	5.4 (0.70)
*P* value ^1^	0.41	0.35	0.87
Insulin µU/ml			
Week 0	8.7 (7.10)	10.1 (6.20)	11.2 (6.17)
Week 12	9.6 (8.00)	8.8 (4.70)	9.9 (5.95)
*P* value ^1^	0.12	0.06	0.054
Triglycerides (TG) mmol/L			
Week 0	1.01 (0.51)	1.10 (0.45)	1.18 (0.38)
Week 12	1.18 (0.58)	1.13 (0.46)	1.19 (0.42)
*P* value ^1^	**0.02**	0.60	0.90
Total cholesterol(TC) mmol/L			
Week 0	4.91 (1.35)	5.21 (1.00)	5.35 (1.19)
Week 12	4.96 (1.33)	4.90 (0.90)	5.29 (1.25)
*P* value ^1^	0.78	**0.01**	0.48
High-Density lipoprotein–(HDL)-cholesterol mmol/L			
Week 0	1.51 (0.42)	1.42 (0.33)	1.51 (0.28)
Week 12	1.49 (0.34)	1.40 (0.34)	1.51 (0.31)
*P* value ^1^	0.57	0.43	0.92
Low Density lipoprotein(LDL)-cholesterol mmol/L			
Week 0	3.05 (0.88)	3.22 (0.59)	3.51 (0.98)
Week 12	2.99 (0.80)	3.06 (0.54)	3.37 (1.06)
*P* value ^1^	0.50	**0.037**	0.31
Non-HDL cholesterol			
mmol/L			
Week 0	3.51 (0.65)	3.72 (0.65)	4.06 (1.22)
Week 12	3.52 (0.57)	3.59 (0.57)	3.92(1.31)
*P* value ^1^	0.98	**0.035**	0.37
Apolipoprotein B48 (apoB48) µg/mL			
Week 0	8.1 (5.0)	6.8 (3.8)	7.5 (6.0)
Week 12	8.7 (5.8)	7.0 (4.6)	6.1 (2.5)
*P* value ^1^	0.31	0.67	0.31
Aspartate aminotransferase (AST) U/L			
Week 0	23.0 (8.1)	24.0 ( 9.9)	24 ± (5.9)
Week 12	23.0 (7.2)	20.0 (5.3)	24 ± 4.63
*P* value ^1^	0.5	**0.014**	0.25
Alanine aminotransferase (ALT) U/L			
Week 0	23.4 (11.2)	22.0 (10.0)	23.7 (11.5)
Week 12	28.4 (16.9)	18.8 (9.5)	18.5 (8.0)
*P* value ^1^	**0.05**	**0.006**	**0.03**

^1^*P* values were derived from mixed-effect models adjusted for age; *p* value of within-group difference between time 0 and week 12. Bold = *p* value < 0.05.
